# Epidemiology of Pediatric Cardiomyopathy in a Mediterranean Population

**DOI:** 10.3390/children11060732

**Published:** 2024-06-15

**Authors:** Alena Bagkaki, Fragiskos Parthenakis, Gregory Chlouverakis, Aris Anastasakis, Ioannis Papagiannis, Emmanouil Galanakis, Ioannis Germanakis

**Affiliations:** 1 School of Medicine, University of Crete, 71 003 Heraklion, Greece; alenabagaki@gmail.com (A.B.); fparth@med.uoc.gr (F.P.); gchlouve@uoc.gr (G.C.); emmgalan@uoc.gr (E.G.); 2 Onassis Cardiac Surgery Center, Syggrou Av. 356, 176 74 Athens, Greece; anastasakis@ocsc.gr (A.A.); papagiannis@ocsc.gr (I.P.)

**Keywords:** cardiomyopathy, childhood, pediatric cardiomyopathy, epidemiology, Crete, Mediterranean

## Abstract

Background. Our knowledge regarding the epidemiology of pediatric cardiomyopathy is based on large national population studies reporting an annual incidence of 1 case per 100,000 children, with a higher incidence observed in infancy and among selected populations. The aim here is to document the epidemiology of pediatric cardiomyopathy in a Mediterranean population. Methods. Children younger than 18 years of age living on the Mediterranean island of Crete, Greece, who have been evaluated since the establishment of tertiary pediatric cardiology services (2002–2022) were included in this retrospective study. Results. A total of 40 children were included, corresponding to an average annual incidence of pediatric cardiomyopathy of 1.59 cases (95% CI: 1.4–2.3) and a prevalence of 26 cases per 100,000 children. In decreasing order of frequency, most cases corresponded to dilated (50%), followed by hypertrophic (42.5%), arrhythmogenic (5%), and restrictive (2.5%) cardiomyopathy. An etiology was identified in 40%, including a genetic diagnosis in 22.5%. Conclusions. The incidence of pediatric cardiomyopathy in the Mediterranean island of Crete is higher compared with that reported previously for other Caucasian populations. Further study is needed to investigate the exact prevalence and specific genetic factors associated with the epidemiology of pediatric cardiomyopathy in Mediterranean populations.

## 1. Introduction

Cardiomyopathies (CMPs) are defined as myocardial disorders that manifest with various structural and functional phenotypes in the absence of other known cardiovascular disorders (congenital, valvular, and coronary artery diseases) [1]. The spectrum of CMPs in children differs from that in adults, being heterogeneous in origin. Although sarcomeric and cytoskeleton mutations can also cause pediatric cardiomyopathies, syndromic, metabolic, or neuromuscular disorders are also relevant [2,3]. The most common morphological phenotypes are dilated (DCM) and hypertrophic (HCM) cardiomyopathy, whereas arrhythmogenic (ACM), restrictive (RCM), and LV non-compaction (LVNC) cardiomyopathy occur less frequently [2].

Our knowledge regarding the epidemiology of pediatric CMPs is based on limited North American [4], Australian [5], and European registries [6]. These population studies and registries estimate the overall annual incidence of primary CMP in children to be around 1 per 100,000 children, with a higher incidence during the first 2 years of life, but ethnic/geographical differences are also present [4,5,6]. Our knowledge about the epidemiology of pediatric CMP in the Mediterranean region is limited. The aim of our study was to determine the prevalence and the annual incidence of pediatric CMP in the pediatric Mediterranean population of the island of Crete. To the best of our knowledge, this represents the first study regarding the epidemiology of pediatric CMPs in the Southeastern Mediterranean region.

## 2. Materials and Methods

This is a retrospective study including children younger than 18 years of age living in the region of Crete Island with the diagnosis of CMP who were presented to the Pediatric Cardiology Unit, University Hospital in Heraklion, Crete, during the 20 years since its first establishment (January 2002–December 2022). The cumulative prevalence at the last year of enrollment and the overall annual incidence rate of pediatric CMP in the pediatric population of Crete Island during the whole period of enrollment was estimated. This study was performed in line with the principles of the Declaration of Helsinki. The approval for collecting and processing the data from the medical records of each study patient was obtained from the University Hospital Heraklion, Ethics Committee, approval number 1007, 33/2.12.2020. Data collection included demographic characteristics, brief family history, clinical findings, and quantitative echocardiographic measurements. All personal data became anonymous.

The classification of CMPs in our study was based on the structural and functional phenotype using a classification based on morphofunctional echocardiographic characteristics [2] and following the European Society of Cardiology approach [1,7]. Clearly defined inclusion morphological criteria in accordance with previous population-based studies on epidemiology of pediatric CMPs [4,5,8] were used for enrollment in our study. Patients with any of the secondary causes of CMPs (congenital heart disease, hypertension, endocrine and pulmonary diseases, toxic exposure, and drugs), the cohorts of asymptomatic pediatric patients with incomplete disease expression (subclinical forms), or children phenotypically negative but genotypically positive were excluded [2,8].

The inclusion criteria were based on echocardiographic measurements of the LV end-diastolic dimension (LVEDD), LV posterior wall and septal thicknesses, LV end-systolic dimension (LVESD), fractional shortening (FS), and ejection fraction (EF). All measurements were expressed as z-scores > 2 standard deviations to adjust for patient size. The Boston Pediatric Echo Z-Score database was used as a reference data resource [9]. The diagnosis of DCM was based on LV end-diastolic diameter (LVEDD) and LV end-systolic diameter (LVESD) greater than 2 standard deviations (SDs) from the mean value for the population corrected for body surface area (z-scores > 2) with decreased systolic function, characterized by reduced measures of FS < 28% and EF < 55%. The diagnosis of HCM was made if the regional or global maximal LV wall thickness was greater than 2 standard deviations above the body-surface-area-corrected mean (z-scores > 2) with both normal and reduced LV systolic function. The diagnosis of RCM was based on specific patterns: both atria enlarged relative to ventricles of normal or small size with evidence of impaired diastolic filling and the absence of significant valvular heart disease. The diagnosis of ACM was based on positive diagnostic criteria. The autopsy report (SCD as a presenting symptom) was considered as an inclusion criterion.

The evaluation of patients included medical history, physical evaluation, standard blood chemical analysis, chest radiography, and echocardiography in all cases. CMR was performed in selected patients. The investigation for inborn errors of metabolism was performed in the majority of patients diagnosed in infancy. Genetic evaluation was performed in a subset of patients. The prevalence and incidence rates were calculated accordingly to the age specific population at risk between 2002 and 2022 on the island of Crete. The pediatric population data were obtained from the Hellenic Statistical Authority Office [10]. The data that support the findings of this study are available from the corresponding author on reasonable request.

Statistical analysis. The incidence rates in various subgroups (year, sex, age, and type of cardiomyopathy) were compared using a Fisher exact probability test and exact 95 percent confidence intervals. The data were summarized as frequencies and percentages for categorical data and as medians and means for the age of presentation. All reported *p* values were based on two-sided tests.

## 3. Results

**Figure 1:**
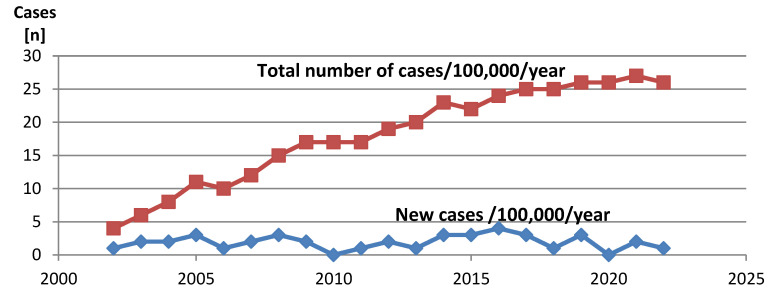
Prevalence and annual incidence of pediatric CMP 2002–2022 in Crete.

Over the period of 20 years (January 2002–December 2022), 40 new cases of pediatric CMP were diagnosed. The total average island population in this period was 614,000, including a total of 119,343 children less than 18 years of age. The average annual incidence of new CMP pediatric cases (<18 yrs) on Crete Island was estimated at 1.59 per 100,000 (95% CI: 1.4 to 2.3). There was no evidence of change over time in the incidence rate of any type of CMP. The cumulative prevalence of pediatric CMP in Crete increased in the first years following the establishment of pediatric cardiology services, reaching a plateau in the last year of enrollment, corresponding to a cumulative prevalence of 26 cases per 100,000 children during the last year of enrollment (Figure 1).

The incidence of DCM was estimated at 0.79/100,000/year, a total of 20 cases of DCM (50%). The incidence of HCM was estimated at 0.67/100,000/year, a total of 17 cases of HCM (42.5%). The incidence of other types of CMP was 0.11/100 000/year, corresponding to 2 cases of ACM (5%) and 1 case of RCM (2.5%) (Table 1).

Of the 40 children with CMP, 27 patients were male (67.5%) and 13 patients were female (32.5%, *p* = 0.001). The association between the type of CMP and sex was significant only in DCM patients (male 80%, *p* < 0.0001). In HCM, male and female distribution was at nearly equal percentages (nine males and eight females). The other CMPs were very rare, and a sex dominance was not observed.

The clinical presentation of CMP was symptomatic in 34 (85%) children (Table 1). Congestive heart failure symptoms were dominant in the clinical symptomatology of 32.5% pediatric CMP patients. Heart failure symptoms were present in all DCM patients diagnosed in infancy (13 infants of the 20 DCM cases). Murmur and exercise intolerance were present in all HCM patients diagnosed in childhood and adolescence (12 children from the 17 HCM cases). Congenital malformations were present in five infants with CMP. Syncope/seizures episodes were presenting symptoms in three children (infant HCM, child HCM, and adolescent ACM). Exercise syncope was an initial symptom in one adolescent with ACM. Sudden cardiac death (SCD) was the first manifestation of ACM in a single adolescent patient, based on autopsy findings. A total of six (15%) children were completely asymptomatic at the time of CMP diagnosis, with four cases (10%) being evaluated during cascade family screening and two cases (5%) during preparticipation screening for sports. The age distribution was not uniform. Proportionally, most cases (20 infants) were diagnosed in the first two years of life (50% of patients). The median age at diagnosis was 2 years old (mean 4.5 years old). One third of the patients (13 children) were diagnosed at school age, while the other 7 patients were diagnosed in adolescence. There was an association between the age of diagnosis and the type of CMP. A total of 13 infants (65% of DCM patients) were diagnosed at <1 year of age (*p* = 0.05). The median age at diagnosis of DCM patients was 6 months of age (mean 2.5 years). In our study, there were two age groups with an increased frequency of HCM diagnosis. The first peak of HCM diagnosis was the first year of life—five cases (29. 5% of total HCM cases); the other peak was late childhood–adolescence—eight cases (47% of total HCM cases). All patients with ACM and RCM were diagnosed in adolescence.

The etiology of CMP patients enrolled in our study was identified in 16 (40%) of 40 children (Table 1). The evaluation for inborn errors of metabolisms was performed in nine cases (22.5% of examined children). A positive result was found in nearly 50% of the evaluated patients. Specifically, it revealed one infant HCM phenotype with glycogen storage disease type 3 and three school-aged children with disorders of fatty-acid metabolism—two children with the HCM phenotype (different families, no relatives) with primary carnitine deficiency and one child with the DCM phenotype with LCAD (long-chain acyl-CoA dehydrogenase deficiency). In our cohort, there was only one patient with the HCM phenotype with a genetically confirmed diagnosis of Noonan syndrome.

Other patients with malformations and/or syndromic clinical features without confirmed genetic diagnosis were not included in this etiologic subgroup. One adolescent DCM patient with the diagnosis of Duchenne’s muscular dystrophy (neuromuscular disease) was enrolled in our study. Acute myocarditis was the initial diagnosis in one child with DCM (the diagnosis was established by CMR imaging). Genetic evaluations were performed in 13 cases (32.5% children), in patients with different forms of CMP. The genetic cause was identified in 29 cases (2.5% children). Positive results, i.e., pathogenic variants, were found in nine cases (69% of those tested). In four cases (30% of those tested), a DNA analysis revealed pathogenic sarcomeric variants: two children with the HCM phenotype (pathogenic variant of the MYH7 gene) and two sibling children with the DCM phenotype, double heterozygous (MYH7/TTN).

The distribution of pediatric CMP cases in Crete is not homogenous, but there is a significant concentration of children with CMP in the western regions of the island. The comparison of the incidence of pediatric CMP on the island of Crete compared to previous studies is presented on Table 2.

The outcome of CMP patients was variable, ranging from radical improvement in one DCM patient to enlistment for heart transplantation in one DCM patient. Medical treatment was indicated in 95% patients. ICDs were implanted to seven patients (four children with HCM, two children with DCM, and one child with RCM). Death was the outcome for five children (12.5% of the total)—two DCM cases, one HCM case, and two ACM cases.

## 4. Discussion

The Mediterranean region is characterized by increased prevalence of many hereditary diseases. Historical populations’ movements that occurred along the Mediterranean Sea in the past had a crucial impact on the variability in prevalence and in the genetic structure of these diseases in different ethnic groups of the Mediterranean population. Hemoglobinopathies and glucose-6-phosphate dehydrogenase (G6PD) deficiency are the most common single-gene disorders encountered in the region [11]. Familial Mediterranean fever (FMF), the most frequent autoinflammatory disease, is common in the Eastern Mediterranean area but rare elsewhere [12]. Increased prevalence of specific hereditary diseases, including inherited CMPs, is typical for historically isolated and differentiated populations living in the Mediterranean islands [13]. Aegean Islands and the island of Crete are geographical regions with increased prevalence of ARVC (Naxos disease) and hereditary transthyretin-related amyloidosis (hATTR) cardiomyopathy [14], respectively. While the epidemiology of adult CMP in the Mediterranean region is described and monitored by European and ethnic registries [15], the knowledge of pediatric CMP epidemiology in the Mediterranean region is limited [16].

We aimed to record the epidemiologic data of pediatric CMPs on the island of Crete and to compare the variability that we found with the results of the other population-based studies [4,5,6]. The island of Crete is a clearly defined geographic area with a mixed population. The pediatric population of Crete, more than 97% Caucasian, partly lives in urban areas but also in relatively isolated rural mountain areas. In these communities, increase rates of parental consanguinity were documented, which may lead to a high prevalence of private and ultra-rare AR disorders [17]. A limited number of children, nearly 3%, belong to other ethnic groups (Arabs and Asians) [10].

Regional differences in the incidence of CMP were found. Τhere was an increased concentration of children with CMP in the central–western rural mountain regions of the island, with the highest incidence of pediatric CMPs in all years of the study (incidence 3.2 per 100,000/years vs. 1.59 per 100,000/year). A similar interesting distribution of genotypes in an east–west direction among native Cretans has been previously described, possibly reflecting the diverse patterns of island colonization in the past from different Mediterranean regions [18].

In comparison with previous studies, the annual incidence of primary CMP was estimated at 1.59 per 100,000 children younger than 18 years of age. There was no significant difference in the frequency of the diagnosis according to the year. The annual incidence in our study was estimated higher than incidences reported in other population-based studies in North America, 1.13/100,000/year [4]; Australia, 1.24/100,000/year [5]; and Finland, 0.65–0.74/100,000/year [6].

The most frequent phenotype in our study was DCM (50%), which correlates with the results of other studies [4,5]. The incidence of DCM was estimated at 0.79 per 100,000 children/year, which correlates with the data from a large prospective study undertaken in the United Kingdom and Ireland [19], where the authors reported an incidence of 0.76 symptomatic DCM cases per 100,000 children under 16 years, which is higher than the data from multicenter North American studies [6,20], where an incidence of 0.57 DCM cases/100,000/year was reported. In the first year of life, 65% of children with DCM were diagnosed, while nearly 75% of them were diagnosed in the first 2 years of life. Congestive heart failure symptoms were dominant in all children <2 years of age diagnosed with DCM. In a multicenter North American study [4] as well as in the National Australian Childhood Cardiomyopathy Study [5], the highest annual incidence of DCM was observed during the first year of life. A Finnish study demonstrated an 11-fold higher incidence of DCM during the first year of life [6].

HCM was the second most frequent type of CMP in our study (42.5%). The incidence of HCM was estimated at 0.67/100,000/year. The pattern of two peaks of increased incidence was typical for patients with HCM. The first early peak of incidence of HCM was in infancy, with the second peak in late childhood and adolescence. This pattern of “two-peak” incidence in HCM cases was presented in reports of other investigators [21]. The data from a multicenter cohort of Marston et al. [22] indicate that cases of phenotypic HCM tends to cluster during infancy and adolescence, including high proportions of patients with RASopathies and inborn errors of metabolism in infancy [23].

The only patient of our study with RCM was diagnosed at an early adolescent age. RCM is a rare form of pediatric CMP and becomes more frequent with increasing age [24], frequently with positive genetic findings [25].

The diagnosis of ACM was established in both patients at early adolescence, confirming our bibliography resources [26]. The phenotypic features of ACM may vary, ranging from asymptomatic to life-threatening ventricular arrhythmias. Both of our patients were symptomatic, but the clinical presentations were diametrically different. Syncope during exercise was an initial symptom in one adolescent patient diagnosed with arrhythmogenic right ventricular cardiomyopathy (ARVC). SCD is suggested to be the first clinical manifestation of the disease specifically in the young people [27], similar to one adolescent patient of our cohort. The diagnosis of ARVC was established by an autopsy report.

Pediatric CMPs are highly heterogeneous in origin. A known or presumed cause of CMP patients enrolled in our study was identified in 40% of children, which correlates with the results of other studies [8,19]. Inborn errors of metabolism were diagnosed in 10% of children (one infant and three school aged children). Metabolic disease evaluation was positive mostly in children with the HCM phenotype (23.5% of all HCM patients), but inborn error of metabolism was diagnosed in only patient with the DCM phenotype. Similar results were reported in The Pediatric Cardiomyopathy Registry of children diagnosed with HCM, where 8.7% had inborn errors of metabolism [21]. In the study of Kindel et al. [28], significant rates of metabolic causes in children with CMP (19%) were identified, confirming the importance of a broad differential for the clinical evaluation of children.

The progress in understanding the genetic base of CMP has shown that the genetics of CMPs in children is extremely heterogeneous and complex [17]. Genetic variants causing CMP in children can be isolated (merely cardiac involvement) or they can have systemic features (multiorgan involvement). The RASopathies are the most well-known syndromic causes of pediatric cardiomyopathy [17,28]. In our cohort, there was only one patient with a genetically confirmed diagnosis of Noonan syndrome and the HCM phenotype. The most common neuromuscular disorders with cardiac manifestations are the muscular dystrophies. The type and extent of cardiac manifestations are specific to the type of neuromuscular disorder. The most common cardiac findings include the DCM or HCM phenotype [29]. One adolescent patient with a diagnosis of Duchenne’s muscular dystrophy and the DCM phenotype from the pediatric neuromuscular disorder cohort was enrolled. Acute myocarditis was the initial diagnosis in one child with DCM (the diagnosis was established by CMR imaging). Viral myocarditis is the most common cause of inflammatory DCM in children [2]. The results from the endomyocardial biopsy are still the reference standard for diagnoses of myocardial inflammation. Given the difficulties of proving inflammation in the myocardium, the clinical diagnosis is based on non-invasive diagnostic methods (CMR imaging) [2].

Genetic evaluation was performed in nearly one third of cases (32.5%) of all CMP patients. DNA analyses were positive in 22.5% of children. Genetic testing revealed a pathogenic variant in 69% of the evaluated children. Variants in genes encoding sarcomeric proteins made up 45% of these pathogenic variants. These results of our patients’ DNA analyses are similar to those found in previous studies. Pediatric CMPs are genetically heterogeneous, with many different causative genes and multiple mutations in each gene. Mutations in genes encoding sarcomeric proteins are the most common abnormalities in children with isolated HCM [1], but they are also associated with other CMPs [30]. Desmosomal gene variants are associated with ACM [2,17].

The present study findings should be viewed by taking into account the study limitations, including the retrospective study design, the small sample size of the reference population, and the limited diagnostic evaluation regarding underlying etiology (genetics) due to its associated cost.

## 5. Conclusions

The incidence of pediatric CMP in Crete is higher than that in other population-based studies, manifesting also regional clusters mainly in the isolated rural and mountain western regions. The types and age distribution of pediatric CMP cases was similar to that previously described. DCM was the most common type in our study, accounting for 50% of all CMP patients. Τwo-thirds of the cases were diagnosed in the first 2 years of life, with dominant heart failure symptomatology. The clinical presentation at diagnosis was asymptomatic in 10% patients only. HCM was the other most common type, accounting for 42.5% patients. One-third of the cases were diagnosed in infancy based on various clinical signs and symptoms, except for heart failure symptoms. Two-thirds of the cases were diagnosed in childhood and adolescence. Asymptomatic presentation was typical for patients diagnosed during cascade family screening or preparticipation sports screening. ACM and RCM are rare types of CMP, and their diagnoses were established in all cases in adolescence. Clinical presentation of both ACM patients was fatal, due to sudden cardiac death and life-threatening arrhythmia, respectively. An etiology was identified in 40%, being genetic in 22.5% of cases. Further studies are needed to investigate whether Mediterranean populations are at increased risk of pediatric CMP and for the underlying genetic factors of any differences.

## Figures and Tables

**Table 1:** Epidemiology of pediatric CMP in Crete 2002–2022.

	DCM n (%)	HCM n (%)	ACM n (%)	RCM n (%)	CMP n	CMP %
**Patients**	20 (50)	17 (42.5)	2 (5)	1 (2.5)	40	100
Male	16 (80)	9 (53)	1 (50)	1(100)	27	67.5
Female	4 (20)	8 (47)	1 (50)	0	13	32.5
**Age at diagnosis**						
<1 year	13 (65)	5 (29.5)	0	0	18	45
2–11 years	7 (35)	4 (23.5)	0	0	11	27.5
12–18 years	0	8 (47)	2 (100)	1 (100)	11	27.5
**Clinical presentation at diagnosis**						
**Clinical symptoms**						
Heart failure symptoms	13 (65)	0	0	0	13	32.5
Murmur	2 (10)	6 (35)	0	0	8	20
Congenital malformations	0	5 (29)	0	0	5	12.5
Exercise intolerance	2 (10)	2 (12)	0	0	4	10
Syncope/Seizure episodes	0	2 (12)	1 (50)	1 (100)	4	10
Myocarditis	1 (5)	0	0	0	1	2.5
Sudden cardiac death	0	0	1 (50)	0	1	2.5
**Asymptomatic**						
Cascade family screening	1 (5)	3 (17)	0	0	4	20
Preparticipation sports screening	1 (5)	1(6)	0	0	2	5
**Specific primary causes of CMP**						
**Genetic causes**						
Pathogenic sarcomeric variant	0	4 (23)	0	0	4	10
Pathogenic non-sarcomeric variant	3 (15)	2 (12)	0	0	4	10
**Inborn errors of metabolism**						
Glycogen storage disease type 3	0	1 (6)	0	0	1	2.5
Primary carnitine deficiency (disorders of fatty-acid metabolism)	0	2 (12)	0	0	2	5
LCAD (disorders of fatty-acid metabolism)	1 (5)	0	0	0	1	2.5
**Neuromuscular disorders**						
Duchenne’s muscular dystrophy	1 (5)	0	0	0	1	2.5
**Inflammatory causes**						
Acute myocarditis	1 (5)	0	0	0	1	2.5
**Malformation syndromes**						
Noonan syndrome	0	1 (6)	0	0	1	2.5

Abbreviations: CMP—cardiomyopathy, DCM—dilated cardiomyopthy, HCM—hypertrophic cardiomyopathy, ACM—arrhythmogenic cardiomyopathy, RCM—restrictive cardiomyopathy, LCAD—long-chain acyl-CoA dehydrogenase deficiency.

**Table 2:** Characteristics of pediatric CMP epidemiologic studies.

	Arola et al. 1997 [6]	Nugent et al. 2003 [5]	Lipshulz et al. 2003 [4]	Present Study
Pro/ retrospective	retrospective	retrospective	prospective	retrospective
Duration (years)	12 (1980–1991)	10 (1987–1996)	3 (1996–1999)	20 (2002–2022)
Country	Finland	Australia	USA	Greece- Crete
Ages	0–20	0–10	0–18	0–18
Cases	118	314	467	40
CMP incidence/ 100,000/year	0.65–0.74	1.24	1.13	1.59
95% CI	0.65–0.74	1.11–1.38	1.03–1.23	1.4–2.3
HCM (%)	37	25	42	42.5
DCM (%)	52	58	51	50

Abbreviations: CMP—cardiomyopathy, DCM—dilated cardiomyopathy, HCM—hypertrophic cardiomyopathy.

## Data Availability

The data presented in this study are available from the corresponding authors upon request. The data are not publicly available due to privacy and ethical restrictions.
